# Analysis of the Airway Microbiota of Healthy Individuals and Patients with Chronic Obstructive Pulmonary Disease by T-RFLP and Clone Sequencing

**DOI:** 10.1371/journal.pone.0068302

**Published:** 2013-07-09

**Authors:** Tetyana Zakharkina, Elke Heinzel, Rembert A. Koczulla, Timm Greulich, Katharina Rentz, Josch K. Pauling, Jan Baumbach, Mathias Herrmann, Christiane Grünewald, Hendrik Dienemann, Lutz von Müller, Robert Bals

**Affiliations:** 1 Department of Internal Medicine V – Pulmonology, Allergology, Respiratory Intensive Care Medicine, Saarland University Hospital, Homburg, Germany; 2 Institute of Medical Microbiology and Hygiene of the University of Saarland, Homburg/Saar, Germany; 3 Clinic of Pneumology, University Hospital Giessen and Marburg, Philipps-University Marburg, Marburg, Germany; 4 Department of Computational Systems Biology, Max-Plank Institute for Informatics, Saarbruecken, Germany; 5 Department of Thoracic Surgery, Thoraxklinik at the University Hospital Heidelberg, Heidelberg, Germany; University of Tübingen, Germany

## Abstract

Chronic obstructive pulmonary disease (COPD) is a progressive, inflammatory lung disease that affects a large number of patients and has significant impact. One hallmark of the disease is the presence of bacteria in the lower airways. Objective: The aim of this study was to analyze the detailed structure of microbial communities found in the lungs of healthy individuals and patients with COPD. Nine COPD patients as compared and 9 healthy individuals underwent flexible bronchoscopy and BAL was performed. Bacterial nucleic acids were subjected to terminal restriction fragment (TRF) length polymorphism and clone library analysis. Overall, we identified 326 T-RFLP band, 159 in patients and 167 in healthy controls. The results of the TRF analysis correlated partly with the data obtained from clone sequencing. Although the results of the sequencing showed high diversity, the genera *Prevotella*, *Sphingomonas*, *Pseudomonas*, *Acinetobacter*, *Fusobacterium*, *Megasphaera*, *Veillonella*, *Staphylococcus*, and *Streptococcus* constituted the major part of the core microbiome found in both groups. A TRF band possibly representing *Pseudomonas* sp. monoinfection was associated with a reduction of the microbial diversity. Non-cultural methods reveal the complexity of the pulmonary microbiome in healthy individuals and in patients with COPD. Alterations of the microbiome in pulmonary diseases are correlated with disease.

## Introduction

Chronic obstructive pulmonary disease (COPD) is a respiratory disease associated with chronic inflammation of the lung leading to tissue destruction and emphysema. Bacterial colonization is one critical factor contributing to COPD progression and the occurrence of acute exacerbations (AECOPD) [1], [2]. The “microbial hypothesis” highlights a vicious cycle, in which smoking results in impaired innate lung defense with subsequent changes in the pulmonary microbiome. This chronic colonization and infection results in further impairment of the mucociliary clearance and host defense apparatus due to aberrant mucus secretion, disrupted ciliary activity, and airway epithelial injury. Multiple pathways have been described how smoke exposure destroys lung host defense mechanisms, including the suppression of antimicrobial peptides [3], the destruction of the epithelial barrier [4], and the colonization of lower airways with tobacco-associated microbes [5].

Microbiological studies in COPD have been limited to the detection of specific respiratory pathogens such as *P. aeruginosa*, *M. catarrhalis,* and *H. influenza* [6], [7] by means of cultural methods or pathogen-specific PCR. The application of culture independent methods to detect the presence of microorganisms has been introduced to characterize the microbiome of various organs [8]. These methods include real time PCR, sequencing, and restriction fragment length polymorphism analysis [9]. The microbiome of the lungs in patients with cystic fibrosis were characterized by culture independent methods and revealed a complex structure of the microbial community in this disease [10]–[12]. Bacteria found in respiratory tract may represent about 6% of total human microbiome, which is comparable to the skin [13]. Several studies in this field highlight that pulmonary microbial communities are complex and diverse [14]–[18]. These studies focused on different patients groups and used different methodology. Huang et al. in their culture-independent survey focused on the microbiota during COPD exacerbations [16], Hilty et al. studied bacteria presenting in bronchoalveolar lavages of asthmatic children [15]. Differences between these studies indicate a wide interindividual variation and the need of standardization of sampling and analysis procedures. Many recent studies that characterize microbial communities use approaches, which are based on the sequence analysis of the 16S rRNA gene [19]–[22].

The aim of the current study was to examine the pulmonary microbial communities in COPD patients as compared to healthy individuals. Terminal restriction fragment length polymorphism (T-RFLP) analysis and molecular cloning with sequencing were used to characterize the pulmonary microbiome in health and disease.

## Materials and Methods

### Participant Selection

The study was approved by the ethic committees of the University of Marburg and the Landesärztekammer of the Saarland; all participants gave written informed consent. Healthy individuals were defined as never smokers, without chronic disorders and without any respiratory illnesses over one year prior the study. COPD patients had clinically diagnosed COPD as described in the GOLD guidelines (http://www.goldcopd.org/). The diagnosis of COPD was based on patient history, pulmonary function tests, and a post bronchodilator ratio of forced expiratory volume in 1 s (FEV_1_)/vital capacity (VC) or forced vital capacity (FVC) below 0.7. The patient was defined as stable if there was no exacerbation for the previous 4 wks and the patient was not currently having an exacerbation. Table 1 summarizes the characteristics of the study populations.

**Table 1:** Patient and control individuals characteristics; all individuals were caucasian; FEV1 = forced expiratory volume in 1 s, VC = vital capacity, FEV1% = FEV1 predicted, PY = pack years, ICS = inhaled corticosteroid, LABA = long acting beta agonist, LAMA = long acting muscarinic agonist, BAL cells = bronchoalveolar lavage cell number×10 ^5^/ml.

	Age	Sex	FEV1/VC	FEV1%	PY	ICS	LABA	LAMA	BAL cells
COPD 1	60	m	62	61	80	–	–	–	3.5
COPD 2	62	f	47	47	30	–	–	+	3
COPD 3	57	f	65	69	20	–	–	–	10.4
COPD 4	73	m	56	51	47	+	+	–	4.8
COPD 5	67	m	60	58	40	–	–	+	8
COPD 6	69	f	59	57	98	–	–	–	3.7
COPD 7	58	m	41	34	70	+	+	+	6.9
COPD 8	54	f	33	22	60	–	–	+	17.9
COPD 9	67	m	69	48	40	–	–	+	2,1
COPD average (+/− SD)	63±6.3	55.6% male	54±11	49±11	53±25	–	–	–	6.7±2.3
Healthy average (+/− SD)	27±2.7	55.6% male	82±6	98±10	0	–	–	–	0.6±0.1

Bronchoscopy was performed using a flexible video-bronchoscope following mild sedation and local anesthesia with xylocaine 2%. BAL of a segment of the middle lobe or the lingula was done by instillation of 5 times 20 mL of sterile saline and recovered fluid was immediately sent to the research laboratory.

### DNA Purification

BAL samples were stored on ice prior to the DNA extraction. After centrifugation at 2500 rpm at 4°C for 10 min, the supernatant was discarded and the pellet was used as a starting material for the DNA isolation. Total DNA was purified by using a combination of a bead-beating method with the standard phenol:chloroform extraction. Each cell pellet was resuspended in 750 µl of sodium phosphate buffer (112.87 mM Na_2_HPO_4_, 7.12 mM NaH_2_PO_4_) and 250 µl of TNS solution (500 mM Tris-HCl, 100 mM NaCl, 10% SDS (w/v). Tubes were filled with 0.7 g of 0.5 mm sterile zirconia beads (Carl Roth GmbH, Karlsruhe, Germany) and vortexed for 45 sec at 6.5 m/s using the homogenizer Precellys® 24 (PEQLAB Biotechnologie GmbH, Erlangen, Germany). Following chilling on ice and pelleting of remaining cell fragments, lysates were denaturated with one volume of phenol:chloroform:isoamylalcohol (25∶24:1). After short spinning, 800 µl of supernatant were mixed with an equal volume of chloroform:isoamylalcohol (24∶1). Then 650 µl of the DNA containing supernatant were mixed thoroughly. The extracted DNA was precipitated by spinning for 80 min at 4°C with two volumes of PEG 6000 and washed in ice-cold 70% ethanol. Finally it was eluted in 50 µl of EB buffer (QIAGEN GmbH, Hilden, Germany) and additionally incubated at 37°C for 2 h to enhance the DNA recovery yield.

### PCR Amplification of 16S rRNA Genes

Total DNA (100 ng) was used as a starting material for the PCR-based amplification of 16S rRNA gene. The used primer set was: (Ba27f, 5′ - aga gtt tga tcc tgg ctc ag - 3′ and Ba907r, 5′ - ccg tca att cct ttr agt tt - 3′ [23] (Metabion GmbH, Martinsried, Germany) generated a 900 bp long PCR product from *E. coli* DH5α 16S rRNA gene. After initial denaturation at 94°C for 3 min, PCR was performed using 40 cycles of denaturation at 94°C for 30 s, annealing at 50°C for 45 s, and extension at 72°C for 1 min and 30 s, followed by a final extension step at 72°C for 7 min (MyCycler Thermal Cycler, Bio-Rad, Munich, Germany). Total *E. coli* DH5α DNA (100 ng) was used as a positive control and 5 µl of destilled water as a negative control. PCR was run in duplicate and amplified products were pooled, purified using with NucleoSpin Extract II kit (MACHEREY-NAGEL GmbH & Co. KG, Dueren, Germany), and analyzed by electrophoresis using a 1.5% agarose gel.

### T-RFLP Analysis

PCR was performed using a FAM (6-carboxyfluorescein)-Ba27 forward primer (Metabion GmbH, Martinsried, Germany). PCR products were cut out from the agarose gel, purified, and subjected to *Msp*I (Promega GmbH, Mannheim, Germany) digestion for 3 h at 37°C in the dark. Then samples were subjected to capillary electrophoresis on ABI 310 Genetic Analyzer (Applied Biosystems Deutschland GmbH, Darmstadt, Germany) using the GeneScan™ 500 ROX™ XL size standard (Applied Biosystems Deutschland GmbH, Darmstadt, Germany). Data were analyzed with GeneMapper® Software (Applied Biosystems Deutschland GmbH, Darmstadt, Germany). For each sample analysis was done in duplicate.

Terminal restriction fragments (T-RFs) between 50 and 700 bp were included in the analysis and differences in TRF length of ±1 bp in different profiles were considered as identical in order to compare T-RFLP profiles between different samples. The relative abundance of each TRF within a given T-RFLP pattern was calculated as the peak height of the respective TRF divided by the total peak height of all TRFs detected. We compared TRFs with the good quality reference sequences from the RDP database. As additional control we compared the obtained data with those from species from the oral cavity. This comparison resulted in phylogenetic assignment on the genus level of fragments and was performed using a web - based tool Microbial Community analysis III (MiCA 3MiCA 3) with the following parameters: T-RFLP analysis (PAT+), digest sensitivity – 2 mismatches within 2 bases from 5′ of forward primer, window size – forward match ±1 bp. Some fragments could not be matched and assigned because of gaps in the database. Some fragments were identified as “uncultured bacteria” and could not be phylogenetically assigned. The copy number of 16S rRNA genes vary between different species (1 to 15 copies) and thus do not correspond necessarily to number of bacterial cells [24]. All terminal fragments were classified as “of the most frequent occurrence fragment” if the relative fluorescence of peaks was >3%. Microbial diversity was estimated with the Shannon-Wiener diversity index (H’) and species richness value using the Excel software (Microsoft, USA). The Shannon index enables to translate the T-RFLP data (the number and intensity of bands on a gel) into numerical data points that can be analyzed, modeled, and evaluated using various statistical parameters. The species richness value ignores the relative abundance of each phylotype and instead focuses only on the total number of unique phylotypes (bands on the gel) for a particular population [25].

### Cloning and Sequence Analysis

To construct the clone libraries from BAL samples from 18 studied participants, amplicon pools were ligated and cloned using the standard protocol from the pGEM®-T Easy Vector System (Promega GmbH, Mannheim, Germany). 50±10 clones per clinical sampled were subjected to sequencing. Individual cloned 16S rRNA gene sequences were first amplified using the M13F and M13R primers and then sequenced with the 27F primer using an ABI3700 (Applied Biosystems, Inc.). Approximately 900 bp of the sequence (5′–3′, with primer ends) were compared with those available in the GenBank of the National Center for Biotechnology Information (NCBI) by BLAST search. Identification of the species level was defined as ≥98% similarity of the 16S rRNA gene sequence to the sequence of its closest bacterial relative in the GenBank database using online software BlastN (NCBI, USA). After primer, vector fragments, and chimeric sequences were removed (using RunVecScreen and Black Box Chimera Check (B2C2) software respectively), the operational taxonomic units (OTUs) were defined. OTU cutoff was less than 0.03, which corresponded to the strain level [26]. To determine the distances between aligned sequences the distance matrix view was generated in Ugene (Unipro Ugene, Novosibirsk, Russia). 16S rRNA gene sequences were aligned using distance neighbor-joining method (Phylip package) of phylogenetic analysis on Ugene software (Unipro Novosibirsk, Russia) and uploaded to the Interactive Tree of Life project for annotation [27]. Bootstrapping was carried out 1000 times to evaluate trees statistically. A web run version of Sequin program (http://www.ncbi.nlm.nih.gov/projects/Sequin/) was used to submit the sequences to the GenBank database. A total of 128 clones were submitted to the GenBank under the accession numbers “JN382472” to JN382540” and “JN378754” to “JN378814”.

### Statistical Analysis

We used the T-RFLP-analyzed data from BAL samples taken from all study participants. A total of 326 distinct peaks were detected. In order to find distinct COPD and healthy microbiome indicators, we applied supervised learning and cross validation using both, R [28] and WEKA [29]. R is a computational environment that provides a broad spectrum of statistical analysis methods while WEKA is a prominent software tool for machine learning consisting various classifiers as well as clustering techniques. Prior to classification, we first removed bands that are were regularly present from samples from the oral cavity (possibly correlating with *Prevotella*, *Veillonella*, *Porphyromonas,* and *Fusobacterium*). Secondly, bands that were evenly detected in healthy and COPD samples were also removed. Afterwards, we used a taxonomic mapping from species to their corresponding genera. This was done to reduce the feature space. Subsequently, a principal component analysis (PCA) was applied using a restrictive variance cutoff of 95%. PCA is generally used for pattern recognition in a data set and can be applied as a filter prior to machine learning, also allowing easier visualization of patterns within high-dimensional data [30]. Finally, significant genera were extracted for classifier training. We used a Naïve Bayes classifier model for classification. Classifier training, testing and validation were done within a cross validation scheme. Two classification techniques were applied. On the one hand we consistently optimized classification results based on the entire data set by iteratively eliminating low significant variables thus creating a robust classification model. Then we applied a simple K-Means clustering, which is a clustering technique to separate a set of observations into clusters based on their means in order to deduce a core set of distinctive genera [31]. On the other hand we selected genera beforehand using a PCA. The retained genera were then used for classifier training.

Furthermore, we specifically analyzed how restriction fragments corresponding to *Pseudomonas* and *Lactobacillus* interact using a PCA but retaining only these fragments. We visualized the PCA results with an R-biplot [32]. Covariance matrices illustrate dependencies which can additionally be used to analyze species interaction. A negative covariance indicates an inverse proportional relationship between two representatives within a microbial community.

## Results

### Restriction Profiles Reveal High Interindividual Variation of the Pulmonary Microbiome

**Figure 1:**
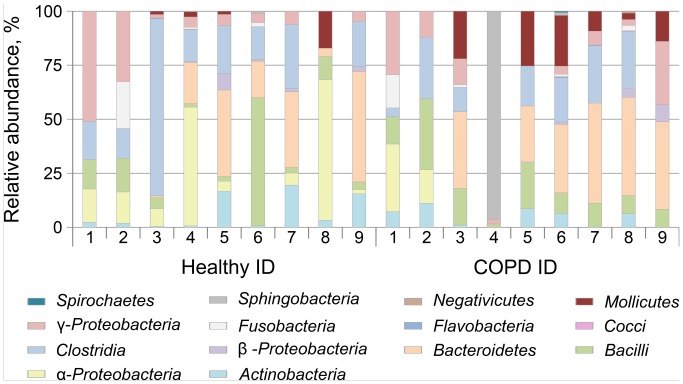
T-RFLP profiles revealed highly interindividual variation of detected bands. Band sizes were correlated with bacterial general, keeping in mind that T-FRLP only allows a prediction but not a determination of the microbial taxa.

A total number of 18 BALs (nine of healthy volunteers and nine of stable COPD patients) served as starting material for PCR amplification of 16S rRNA gene followed by *Msp*I digestion. T-RFLP revealed a total of 326 bands. The mean number of T-RF per individual was 17.7 (±2.32 SEM) in the COPD group and 18.56 (±2.32) in the control group (P = 0.811, two-tailed t test). The range was 12 to 31 (SD 6.95) bands for the healthy individuals and 9 to 27 (SD 6.67) for the COPD patients. Several bacterial species were found consistent with these findings by database searches. However, as T-RFLP results based on PCR with one labeled primer cannot exactly determine the species, such correlations should be viewed with the appropriate caution. All samples showed highly heterogeneous terminal restriction fragment (TRF) patterns (Figure 1).

Matrix tables of the obtained results (data not shown) indicated that some restriction fragments (possibly representing *Bacteroides*, *Lactobacillus*, *Megasphaera*, *Prevotella,* and *Streptomyces*) were most common and appeared in two thirds of healthy individual group samples. In contrast, a different set of restriction bands (possibly representing *Actinomyces*, *Bacillus*, *Corynebacterium*, *Lactococcus*, *Micrococcus*, and *Peptostreptoocccus*) could be found only in certain individuals.

In COPD patients, 4 restriction fragments were present almost in all studied samples, while *9* were distributed irregularly between patients. No COPD-specific pattern could be identified based on restriction fragment analysis. COPD patients with ID numbers 4 and 9 showed very low microbial diversity.

### Statistical Learning Analysis Shows Distinctive Distribution of Restriction Fragments

We detected multiple T-RFPs in each sample group. To determine if any of them are correlated with disease status, a principal component analysis was performed. Classification optimization yielded the results as shown in Table 2. Several specific fragments were used to build a classifier model based on a data set consisting of 9 T-RFLP analyzed BAL samples taken from healthy individuals and COPD patients respectively, correlated with *Neisseria*, *Corynebacterium*, *Staphylococcus*, *Bacillus*, *Mesorhizobium*, *Flavobacterium*, *Lysinibacillus*, *Lachnospiraceae*, *Streptomyces*, *Acinetobacter*, *Megasphaera*, *Pseudomonas*, *Clostridium*, *Sphingomonadales* and *Clostridiaceae*. 16 patients (88.9%) out of 18 were correctly classified into the healthy or COPD group without any false negatives (COPD classified as healthy).

**Table 2:** Confusion matrix of the classification results using a NaiveBayes classifier and cross validation.

	COPD	Healthy
COPD	9	0
Healthy	2	7

With the application of simple K-Means clustering, we could further reduce the selected fragments (retaining bands correlated with *Bacillus*, *Mesorhizobium*, *Flavobacterium*, *Streptomyces*, *Megasphaera,* and *Clostridiaceae*). Given the TN (true negative) and FN (false negative) rates of 0.222 and zero respectively, this set of bands is reliably distinguishing between COPD patients and healthy individuals. One fragment (possibly representing *Mesorhizobium*) was only detected in samples of healthy individuals. Classifier training solely based on a prior feature selection via PCA (including retransformation) did not yield a good learning model and classification performance was non-distinctive. Different variance proportion cutoffs did not significantly change the results.

**Figure 2:**
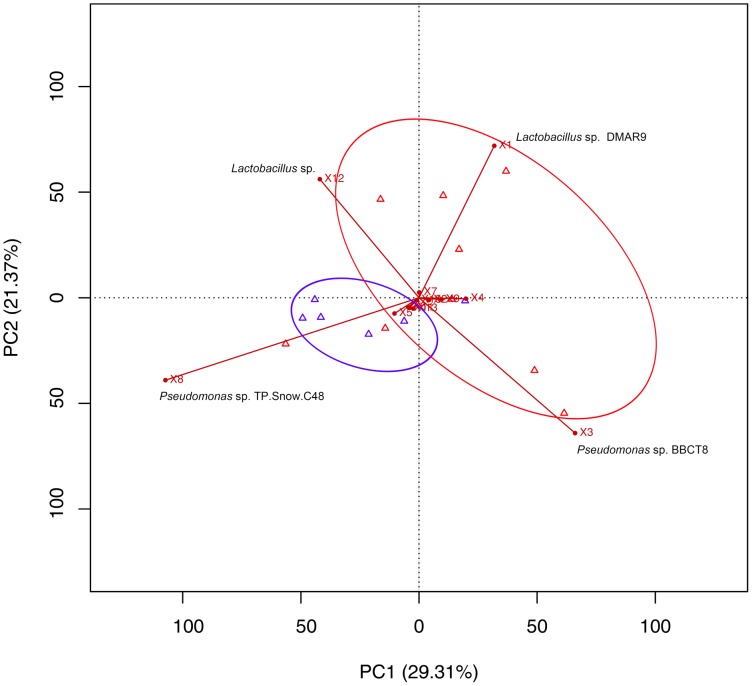
Biplot of the principal component analysis of the bacterial communities from bronchoalveolar lavages samples of the chronic obstructive pulmonary disease patients and healthy individuals. The terminal restriction fragment lengths (in base pairs) corresponding to the correlated bacterial genera are specified in the brackets.

Two bands consistent with the presence of *Pseudomonas* and *Lactobacillus* were used in the PCA analysis to create a biplot using the R library (Figure 2) that revealed the presence of clusters representing data sets from healthy individuals and COPD patients.

**Figure 3:**
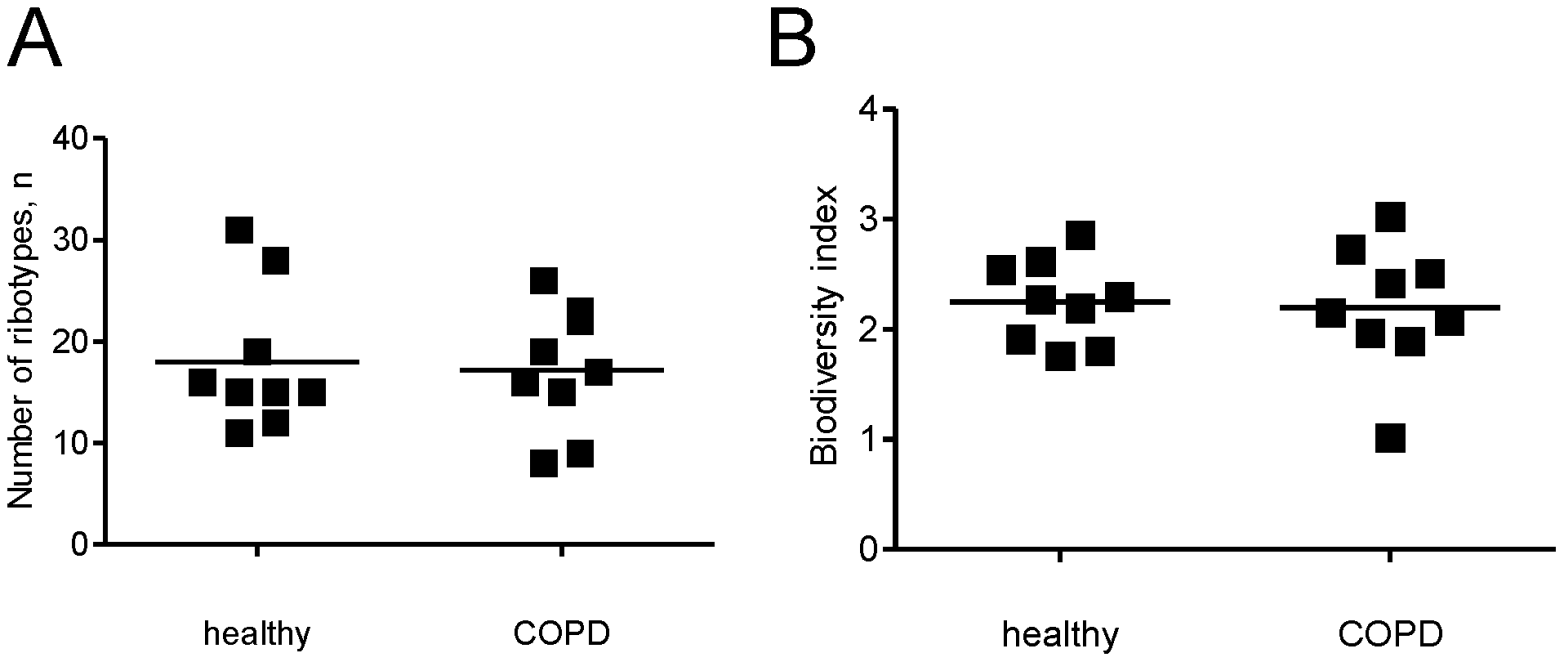
Sample richness (a) and Shannon diversity index (b) based on fragment lengths. Terminal restriction fragments shorter than 50 bp were excluded from the analysis. Empty squares correspond to healthy individuals, filled squares to COPD patients.

In order to quantify the differences in community structures of the two studied groups, the fragment richness and Shannon diversity index were calculated. No difference between COPD patients and healthy individuals could be seen (Figure 3).

### Sequencing Data Revealed the Presence of Distinct Core Genera in Health and Disease

**Figure 4:**
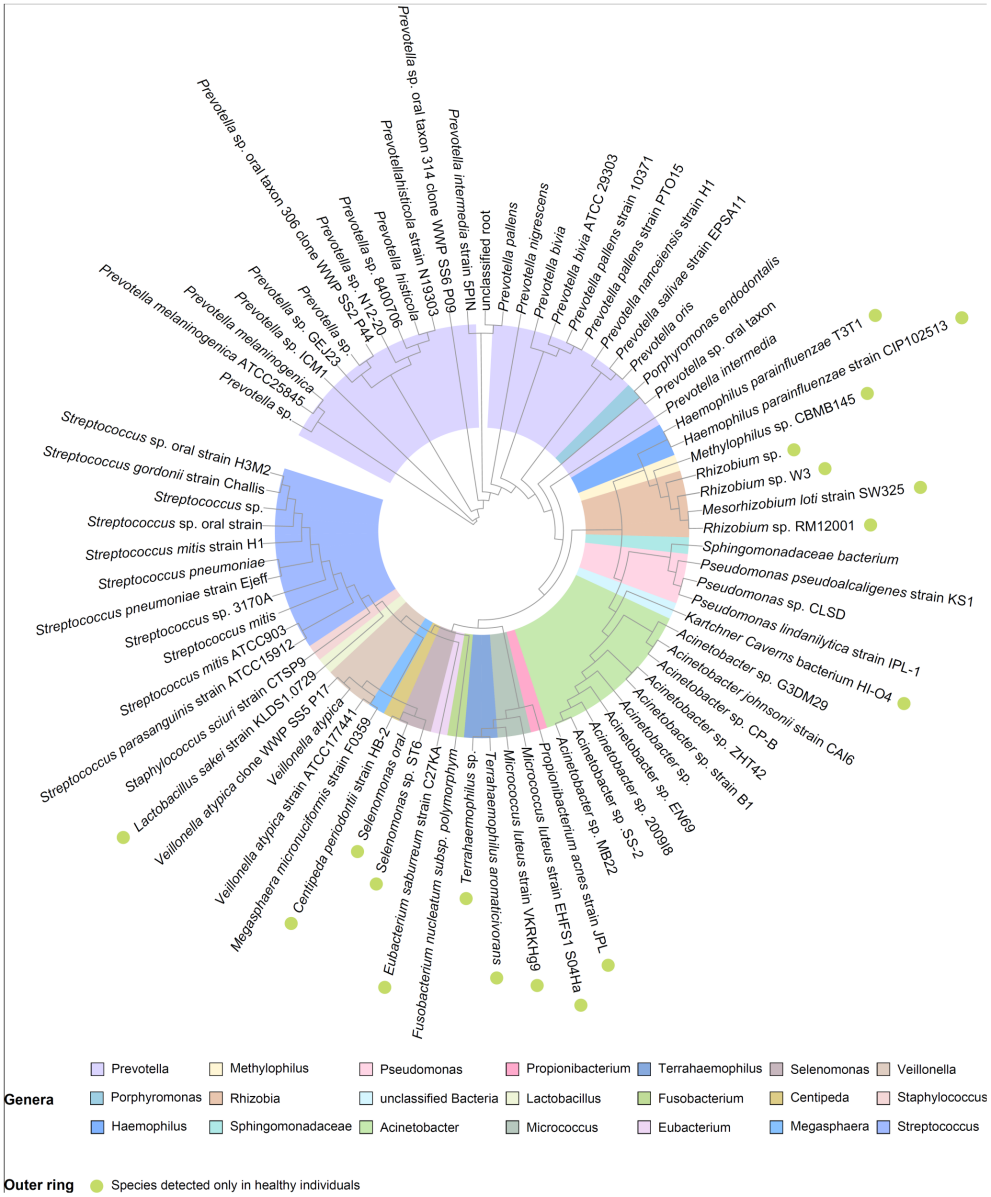
A phylogenetic tree obtained from sequencing analysis illustrates the standard microbiota present in healthy lungs containing 75 leafs. Different genera are denoted with different colors with one exception: the family *Sphingomonadaceae* represents the taxon of higher rank. Bacteria marked with green circles were detected only in healthy individuals, but not in COPD patients.

**Figure 5:**
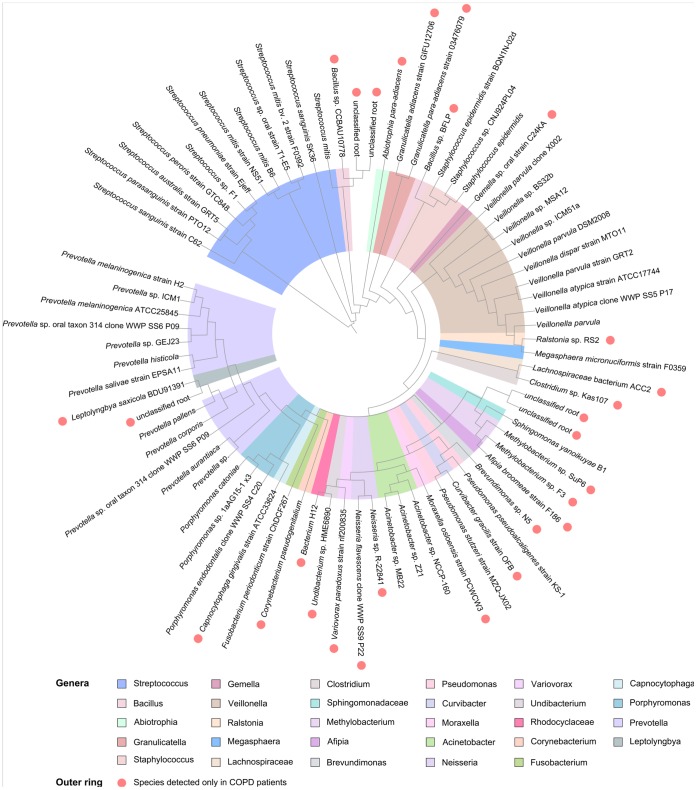
A phylogenetic tree obtained from sequencing analysis illustrates the microbial community of lungs of patients with stable COPD and contains 76 leafs. Different genera are denoted with different colors with two exceptions: the families *Rhodocyclaceae* and *Lachnospiraceae* represent the taxa of higher rank. Bacteria marked with red circles were detected only in COPD patients, whereas other microbes are the part of a core lung microbiome.

A total of 806 clones from all individuals were sequenced and the obtained results were used for database searches. Most of the sequences matched with the phyla *Firmicutes* and *Proteobacteria* (Figures 4, 5) that represented 40 and 35% of the sequences in healthy individuals and COPD patients, respectively. Other sequences matched to the phyla *Fusobacteria*, *Bacteroidetes*, and *Actinobacteria*. The phylum *Cyanobacteria* was characteristic for the COPD patient group. Interestingly, anaerobic representatives of the standard microbiota made up 40% of total community in healthy individuals, and about 34% in COPD patients. Almost 20% of identified bacteria can tolerate oxygen in both sample types. One third of bacterial representatives have a Gram-negative type of cell wall.

Phylogenetic trees were constructed using the clustering neighbor-joining method. After construction, the reliability of trees was estimated by nonparametric bootstrapping. The genera *Prevotella*, *Streptococcus*, and *Acinetobacter* were more diverse, representing multiple species and strains. Three representatives of this genus were detected only in healthy individuals. *Haemophilus parainfluenzae*, *Propionibacterium acnes*, *Micrococcus luteus*, *Terrahaemophilus* sp., *Eubacterium saburreum*, *Selenomonas* sp., *Centipeda periodontii*, and *Lactobacillus sakei* made up 24% of total microbial community and were characteristic for healthy individuals (Figure 4).

A second distance-based neighbor-joining tree was constructed with 76 phylotypes detected in COPD patient group (Figure 5). Twenty nine distinct phylotypes were detected in the COPD patients. The genera *Streptococcus*, *Veillonella,* and *Prevotella* were most common and had the highest diversity. The diagram depicts that there were more than 20 unique species (composing 36.8% of total community) characteristic for COPD state. Unique genera included *Afipia*, *Brevundimonas*, *Curvibacter*, *Moraxella*, *Neisseria*, *Undibacterium*, *Corynebacterium*, *Capnocytophaga*, and *Leptolyngbia*.

A common core microbial community of 9 bacterial genera was identified in all samples analyzed. It included members of *Acinetobacter, Fusobacterium, Megasphaera*, *Prevotella*, *Pseudomonas*, *Sphingomonas*, *Staphylococcus*, *Streptococcus,* and *Veillonella*. We also examined sequencing data for the presence of atypical bacteria (*Mycoplasma pneumoniae*, *Legionella pneumophila*, and *Chlamydophila pneumoniae)*, which are associated with COPD exacerbations [33]. Neither was detected by this method, although *Mycoplasma* and *Legionella* related species were identified in several samples using T-RFLP approach. The results from the RFLP analysis were correlated with the data from sequencing and showed that the results from both methods correlate partly (Table 3).

**Table 3:** An overview of the bacterial genera detected by sequencing and terminal restriction fragment length polymorphism analysis in COPD patients and healthy individuals.

COPD			Healthy		
T-RFLP		Sequencing	T-RFLP		Sequencing
Acinetobacter	X	Acinetobacter	Acinetobacter	X	Acinetobacter
Actinomyces					Actinomyces
			Abiotrophia		
			Afipia		
Alkaliphilus					Alkaliphilus
Bacillus			Bacillus	X	Bacillus
Bacteroides					Bacteroides
			Brevundimonas		
			Capnocytophaga		
		Centipeda			
Clostridium			Clostridium	X X	Clostridium
Corynebacterium			Corynebacterium		Corynebacterium
			Curvibacter		
		Eubacterium			
Flavobacterium					Flavobacterium
Fusobacterium	X	Fusobacterium	Fusobacterium	X	Fusobacterium
Gemella			Gemella	X	Gemella
			Granulicatella		
		Haemophilus			
Lactobacillus	X	Lactobacillus			Lactobacillus
Lactococcus					
Legionella					Legionella
			Leptolyngbya		
Lysinibacillus					Lysinibacillus
Megasphaera	X	Megasphaera	Megasphaera	X	Megasphaera
Mesorhizobium					
		Methylophilus			
			Methylobacterium		
Micrococcus	X	Micrococcus			
			Moraxella		
Mycobacterium					Mycobacterium
Mycoplasma					Mycoplasma
Neisseria			Neisseria	X	Neisseria
Peptostreptococcus					Peptostreptococcus
Porphyromonas	X	Porphyromonas	Porphyromonas		
Prevotella	X	Prevotella	Prevotella	X	Prevotella
		Propionibacterium			
Pseudomonas	X	Pseudomonas	Pseudomonas	X	Pseudomonas
Ralstonia			Ralstonia	X	Ralstonia
Rhizobium	X	Rhizobium			
			Rhodocyclaceae		
Selenomonas	X	Selenomonas			Selenomonas
Sphingobacterium					Sphingobacterium
Sphingomonas	X	Sphingomonadaceae	Sphingomonadaceae	X	Sphingomonas
Staphylococcus	X	Staphylococcus	Staphylococcus	X	Staphylococcus
Stenotrophomonas					
Streptobacillus					Streptobacillus
Streptococcus	X	Streptococcus	Streptococcus	X	Streptococcus
Streptomyces					Streptomyces
		Terrahaemophilus			
Treponema					Treponema
			Variovorax		
Veillonella	X	Veillonella	Veillonella	X	Veillonella
Xanthomonas					Xanthomonas
			Undibacterium		

Due to technical limitation of T-RFLP analysis with one labeled primer, this method can only predict the phylogenetic ID. Identification of a specific genera by both methods is marked by an “X”.

## Discussion

The results of the present study reveal the presence of highly diverse bacterial communities in the lungs of healthy individuals and COPD patients. The microbiome present in the lungs of COPD patients differs from healthy individuals in the prevalence of genera *Afipia*, *Brevundimonas*, *Curvibacter*, *Moraxella*, *Neisseria*, *Undibacterium*, *Corynebacterium*, *Capnocytophaga*, and *Leptolyngbia*. In two COPD patients, we observed a decrease of the bacterial diversity associated with a band possibly correlated with *P. aeruginosa*.

The core microbiome reported here compares well with those reported by other investigators [14]–[18]. The core microbiome in the lower respiratory tract in the present study comprises *Prevotella*, *Sphingomonas*, *Pseudomonas*, *Acinetobacter*, *Fusobacterium*, *Megasphaera*, *Veillonella*, *Staphylococcus*, and *Streptococcus* comparable to the study of Erb-Downward et al. that included the genera *Pseudomonas*, *Streptococcus*, *Prevotella*, *Fusobacterium*, *Haemophilus*, *Veillonella*, and *Porphyromonas* [14]. In the study of Hilty et al., *Bacteroidetes* (particularly *Prevotella* spp.) was more common in control subjects [15]. Huang et al. emphasized the pathogenic potential of newly identified *B. diminuta*, *A. cryaerophilus*, and *L. interrogans* in exacerbated COPD patients [16]. Moreover, they identified two gastrointestinal-associated species namely *H. cetorum* and *C. mucosalis* in respiratory samples. Anaerobes such as *Prevotella* and *Veillonella* are a component of commensal microbiota of the lung [15]. The core genera pool reported by Huang et al. by application of a high-density microarray [16] was much more diverse (27 classified bacterial families) as compared to the results of the present study. Disease status appears to be associated with reduction of the microbial diversity together with the overrepresentation of specific species. In the present study, two patients with low diversity revealed a restriction fragment correlated with *Pseudomonas* sp. A limited bacterial diversity was generally found for COPD patients [14]. Abundance of *Proteobacteria* was found in asthmatics [15], and members of the *Pseudomonadaceae*, *Enterobacteriaceae*, and *Helicobacteraceae* seemed to play the crucial role in severe exacerbations of COPD [16]. Members of the *Comamonadaceae*, *Sphingomonadaceae*, *Oxalobacteraceae* and other bacterial families were correlated with bronchial hypersensitivity [34]. The differences of age between the two groups studies in the present study might have be a factor that contributed to differences between the microbiomes. Taken together, several studies show that the microbiome of the lung can be analyzed by novel methodologies and is altered in patients with obstructive and inflammatory lung disease, such as asthma, COPD, and CF.

Despite the progress in the field of culture independent analysis of the microbiome, data on the respiratory tract are still limited and the published studies comprise a relatively low number of studied individuals. Different methods are currently being used developed to study the microbiome of the human body. The PhyloChip microarray contains more than 1 million oligoprobes and can differentiate about 60 000 bacterial taxa [6]. Studies using microarray technologies could demonstrate a significant complexity of the pulmonary microbiome (1200 bacterial taxa representing 140 distinct families) [16]. Hilty et al. used 454-pyrosequencing, a method of high-throughput DNA sequencing that allows the analysis of statistically reliable number of reads simultaneously [15]. Analysis of T-RFLPs is commonly used in soil and marine microbiology to evaluate changes in bacterial composition in particular ecological niches. The resolution power of T-RFLP is dependent in the labeling of the primers and the analysis process. As the respiratory microbiome is much more restricted in species diversity as compared to soil or intestinal microbiomes, we used one labeled primer, one restriction nuclease, and the length standard ROX1000 for analysis. Similar approaches were used in related studies using material from patients with cystic fibrosis [35], [36]. The resolution of T-RFLP chromatograms is around 2–5 bp, causing that sometimes separate organisms have similar fragment sizes. Sequencing data showed the prevalence of *Lactobacillus* sp. only in healthy individuals. In the present study, T-FRLP based analysis allowed the identification of bacteria in BAL samples and core community data and the results correlated well with those obtained by Sanger sequencing and database analysis. Based on the multiple methods that are presently used, it is not easy to compare the data from different studies.

The lung microbiome contains a large number of oral microorganisms. Representatives of the common oral microbiome were found in COPD patients and less in healthy individuals [14], [15]. Different reasons might account for this observation: 1) Contamination during the sampling procedure might contribute to the results as the bronchoscope is passed through the oro/nasopharynx. 2) The microbiome of the lung might be related to the microbiome of the upper airways and the oropharynx as small volumes of secretions are continuously microaspirated. This later concept is supported by data that indicate an association of lung disease with oral hygiene [37]. A number of factors could have impacted on the results and limited some aspects of the study. The number of patients in this and other studies on microbiome analysis is still small. Also the age difference between the study and control groups could have caused different results on the pulmonary microbiome. While it is currently not clear how age impacts in the composition of the pulmonary microbiome, the gut microbiome is dependent on age [38], [39].

In conclusion, the microbiome of the healthy lung is complex and differs from the microbiome of the respiratory tracts of COPD patients. Two different methods revealed partly diverse microbial spectra. Increasing knowledge about the pulmonary microbiome will provide the opportunity to understand the role of microorganisms in lung diseases.
